# 2493. Barriers and Facilitators to Hepatitis B Screening, Vaccination, and Treatment Amongst West African Immigrants in The Bronx

**DOI:** 10.1093/ofid/ofad500.2111

**Published:** 2023-11-27

**Authors:** Jared Coe, Jessie Birnbaum, Fatima Omarufilo, Samuel Sigal, Matthew J Akiyama

**Affiliations:** Montefiore Medical Center/Albert Einstein College of Medicine, New York, New York; Albert Einstein College of Medicine, Bronx, New York; Montefiore Medical Center, Bronx, New York; Montefiore Medical Center/Albert Einstein College of Medicine, New York, New York; Albert Einstein College of Medicine, Bronx, New York

## Abstract

**Background:**

There is a high burden of hepatitis B virus (HBV) in West Africa. Over the past 20 years, West African immigration to the U.S. has been increasing, especially to the Bronx. Prevalence of HBV infection in West Africa has been reported to be as high as 5-10%. We sought to understand knowledge and attitudes of and barriers and facilitators to HBV screening, vaccination, and treatment in a cohort of people living with and at risk for HBV.

**Methods:**

We recruited participants through an HBV outreach program (The Starfish Program) which provides free HBV education, screening, and vaccination with a focus on West Africans. We conducted one-on-one in-depth, in-person qualitative interviews with West African immigrants over the age of 18 residing in the Bronx. Our interviews were guided by the modified socioecological model (SEM), which includes individual, social network, community, and societal domains. The interviews assessed participants’ understanding of HBV, social issues surrounding HBV in their community, and structural factors affecting their ability to seek healthcare. Data were analyzed in an iterative process using a thematic analysis.

**Results:**

Participants (n=23) had origins in 6 different West African nations, although mainly from Ghana and Nigeria, with a median age of 49.5 (IQR 40-66). 56.5% were female. 7 were HBV surface antigen (HBsAg) positive, 6 were HBsAg negative, and 10 had unknown status at the time of interview. Median time since immigrating was 15.5 years (IQR 4-21). Key barriers included limited knowledge of HBV, HBV-related stigma, lack of health insurance, and undocumented immigration status/fear of deportation. Key facilitators included trust in U.S. healthcare providers and healthcare system, social support networks, and ease of accessing healthcare after obtaining health insurance.

**Figure:**
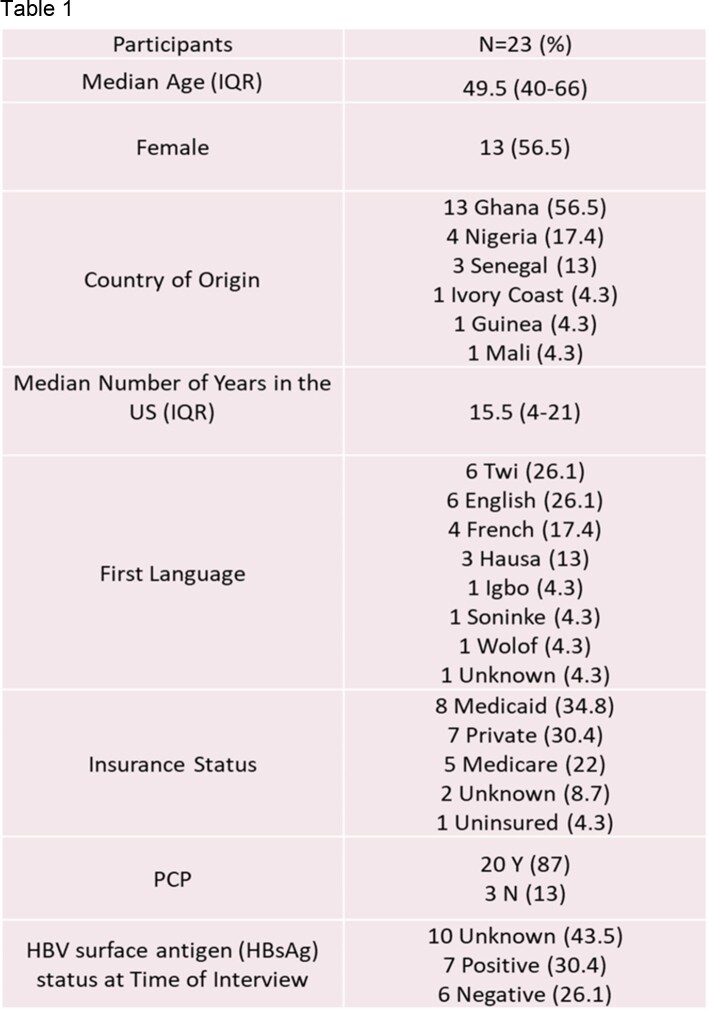

Demographics of interview participants

**Conclusion:**

Raising awareness of HBV, addressing social and structural barriers such as stigma and health insurance, and improving access to culturally sensitive programs among West African communities could help increase HBV screening, vaccination, and treatment in this vulnerable population.

**Disclosures:**

**Samuel Sigal, MD**, Eli Lilly: Grant/Research Support|Gilead Sciences: Grant/Research Support|Gilead Sciences: Speakers Bureau|Intercept: Grant/Research Support|Mallenchrodt: Advisor/Consultant|Mallenchrodt: Grant/Research Support

